# Triboemission of hydrocarbon molecules from diamond-like carbon friction interface induces atomic-scale wear

**DOI:** 10.1126/sciadv.aax9301

**Published:** 2019-11-15

**Authors:** Yang Wang, Naohiro Yamada, Jingxiang Xu, Jing Zhang, Qian Chen, Yusuke Ootani, Yuji Higuchi, Nobuki Ozawa, Maria-Isabel De Barros Bouchet, Jean Michel Martin, Shigeyuki Mori, Koshi Adachi, Momoji Kubo

**Affiliations:** 1 Institute for Materials Research, Tohoku University, 2-1-1 Katahira, Aoba-ku, Sendai 980-8577, Japan.; 2 Department of Mechanical Systems Engineering, Graduate School of Engineering, Tohoku University, 6-6-01 Aramaki-aza-aoba, Aoba-ku, Sendai 980-8579, Japan.; 3 College of Engineering Science and Technology, Shanghai Ocean University, Shanghai 201306, China.; 4 Laboratory of Tribology and System Dynamics, Ecole Centrale de Lyon, 36 Avenue Guy de Collongue 69134, Ecully Cedex, France.; 5 Faculty of Engineering, Iwate University, 4-3-5 Ueda, Morioka, Iwate 020-8551, Japan.

## Abstract

Understanding atomic-scale wear is crucial to avoid device failure. Atomic-scale wear differs from macroscale wear because chemical reactions and interactions at the friction interface are dominant in atomic-scale tribological behaviors, instead of macroscale properties, such as material strength and hardness. It is particularly challenging to reveal interfacial reactions and atomic-scale wear mechanisms. Here, our operando friction experiments with hydrogenated diamond-like carbon (DLC) in vacuum demonstrate the triboemission of various hydrocarbon molecules from the DLC friction interface, indicating its atomic-scale chemical wear. Furthermore, our reactive molecular dynamics simulations reveal that this triboemission of hydrocarbon molecules induces the atomic-scale mechanical wear of DLC. As the hydrogen concentration in hydrogenated DLC increases, the chemical wear increases while mechanical wear decreases, indicating an opposite effect of hydrogen concentration on chemical and mechanical wear. Consequently, the total wear shows a concave hydrogen concentration dependence, with an optimal hydrogen concentration for wear reduction of around 20%.

## Teaser

Triboemission of hydrocarbon molecules such as methane and ethylene induces chemical and mechanical wear of diamond-like carbon.

## INTRODUCTION

Wear of contact materials often causes device failure and is a fundamental problem in science and engineering. With the development of nanotechnology, such as microelectromechanical systems, atomic-scale wear has become increasingly important for the durability and reliability of nanoscale devices. Understanding the processes and mechanisms of atomic-scale wear is an inevitable problem in tribology to avoid device failure and improve durability. However, these processes and mechanisms are still poorly understood because atomic-scale tribological phenomena differ from conventional macroscale tribology (*1*–*3*). For example, chemical reactions and interactions at the friction interface are dominant in atomic-scale tribological phenomena instead of material strength and hardness.

Previous researches on the atomic-scale tribology are mainly focused on friction problems. For example, Song *et al.* (*4*) used atomic force microscopy (AFM) to study the atomic-scale friction of two-dimensional material sheets, and they found a robust superlubricity between graphite and hexagonal boron nitride sheets. Furthermore, molecular dynamics (MD) simulations have been used to investigate the atomic-scale friction behaviors of various materials. Ta *et al.* (*5*) studied the frictional behaviors of iron in a mixed lubrication regime. Klemenz *et al.* (*6*) reported that a single-layer graphene coating reduces the friction on a platinum surface. In addition, we also used MD simulations to reveal the atomic-scale friction processes of diamond-like carbon (DLC), which is one of the most promising solid lubricants (*7*–*10*), and silica (*11*). However, to our best knowledge, few studies focused on atomic-scale wear problems because atomic-scale wear processes involve a series of complicated tribochemical reactions and reaction-induced structural changes. The dynamics of these tribochemical reactions and structural changes are hardly investigated by previous experiments and computer simulations. Recent advances in tribometers, in situ and operando analyzers, and other relevant apparatuses have made the investigation of tribochemical reaction dynamics and structural changes possible. For example, in situ transmission electron microscopy has been used to observe the gradual graphitization of DLC during friction experiments (*12*, *13*). In addition, Gosvami *et al.* (*14*) measured the formation and growth of a tribofilm of a zinc dialkyldithiophosphate antiwear additive from liquid lubricants in situ by using AFM. These structural changes of materials during friction are successfully investigated by the advanced experiment apparatus. Moreover, for the tribochemical reactions, we developed a ball-on-disk tribometer with a quadrupole mass spectrometer (QMS) in a high-vacuum test chamber that is able to detect the detailed number and species of tribochemical reaction products in operando conditions during the friction process. The QMS operando analyzer has been successfully used to elucidate various reactions, such as the tribochemical decomposition of hydrocarbon oils (*15*). Furthermore, advances in high-performance supercomputer systems and high-accuracy reactive MD potentials have allowed large-scale reactive MD simulations to reveal tribochemical reaction dynamics and the mechanisms of structural changes during the sliding of materials. For example, Berman *et al.* (*16*) used reactive MD simulations to reveal the formation mechanism of an onion-like carbon structure from hydrogenated DLC.

In this research, we couple operando friction experiments with reactive MD simulations to clarify the atomic-scale wear and related tribochemical reactions of hydrogenated DLC systematically because the wear mechanisms of DLC have confused people for a long while. Through the operando ball-on-disk friction experiment, we find a previously unidentified atomic-scale chemical wear of DLC that is caused by the triboemission of a variety of hydrocarbon molecules, such as methane, ethane, and ethylene, from the friction interface. Then, by coupling with reactive MD simulations, we demonstrate that the triboemission of hydrocarbon molecules further induces the atomic-scale mechanical wear of DLC. In addition, our simulations reveal that the chemical wear increases and mechanical wear decreases with increasing hydrogen concentration in hydrogenated DLC. Thus, the dominant wear mechanism in hydrogenated DLC changes from mechanical wear to chemical wear as the hydrogen concentration increases. Moreover, chemical and mechanical wear collaboratively result in a concave hydrogen concentration dependence of the total wear, giving an optimal hydrogen concentration of around 20% for achieving the best antiwear properties of DLC.

## RESULTS

### DLC friction experiments

We perform the ball-on-disk friction experiments by using a hydrogen-free DLC-coated Si_3_N_4_ ball sliding against a silicon wafer coated with hydrogenated DLC with a hydrogen concentration of 31%. The whole experiment is conducted in a vacuum for 5 min with a sliding speed of 0.04 m/s under an applied load of 3 N (maximum Hertzian contact pressure of 1.05 GPa). The experimental details are shown in Methods. To clarify the tribochemical reactions and the reaction products at the interface, we place a QMS analyzer in the high-vacuum test chamber. The QMS analyzer detects the fragment ions of molecules based on their mass-to-charge ratio (*m/z*) in operando conditions. During the operando detection by QMS, we interestingly observe that gaseous hydrocarbon molecules are generated and emitted from the friction interface.

**Fig. 1:**
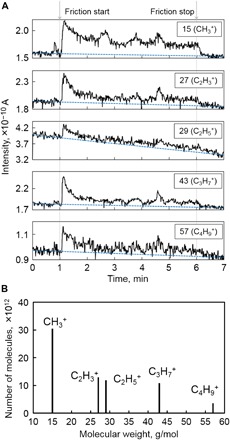
Operando QMS analysis results during the friction experiment of DLC. (**A**) Operando QMS detection results for the fragment ions of methane (CH_3_^+^; *m/z* = 15), ethylene (C_2_H_3_^+^; *m/z* = 27), ethane (C_2_H_5_^+^; *m/z* = 29), propane (C_3_H_7_^+^; *m/z* = 43), and butane (C_4_H_9_^+^; *m/z* = 57) during the friction experiment of DLC. The experiment is conducted from 1 to 6 min. Blue dashed lines indicate the background intensities of each fragment ion in the experiment chamber. (**B**) Molecular weight distribution of hydrocarbon molecules emitted after friction test.

Figure 1A shows the time variation of the intensities of the selected major fragment ions, including methane (CH_3_^+^; *m/z* = 15), ethylene (C_2_H_3_^+^; *m/z* = 27), ethane (C_2_H_5_^+^; *m/z* = 29), propane (C_3_H_7_^+^; *m/z* = 43), and butane (C_4_H_9_^+^; *m/z* = 57), detected by our QMS operando analyzer. The QMS detection results for other fragment ions, such as C_3_H_3_^+^, C_3_H_5_^+^, and C_4_H_7_^+^, are not shown here because they correspond to more than one hydrocarbon molecular type, which is difficult to distinguish only by the *m/z* ratio (e.g., C_3_H_3_^+^ corresponds to propadiene and propyne). At the beginning of the friction test (at 1 min), the intensities of all fragment ions increase sharply, indicating the intense generation and triboemission of the corresponding hydrocarbon molecules from the friction interface during the running-in period. Subsequently, the intensities of all fragment ions decrease gradually and remain almost constant at a level higher than that of the background (blue dashed lines in Fig. 1A), showing that the triboemission of hydrocarbon molecules slows down in the steady-state period. The fast and slow hydrocarbon emissions correspond to the running-in and steady-state periods, respectively. During the running-in period, it is widely accepted that initial asperities of DLC rough surfaces are planarized gradually, leading to the weak collision of surface asperities in the steady-state period. The correlations between the speed of hydrocarbon emission and planarization of surface asperities imply that the triboemission of hydrocarbon molecules is caused by the collision of surface asperities.

Subsequently, to evaluate the hydrocarbon emissions quantitatively, we investigate the number of generated hydrocarbon molecules. The emission rate of each fragment ion is estimated from the intensity changes of the fragment ions detected by QMS (Fig. 1A). (*17*) The details of the method for estimating the emission rate are given in text S1. Thus, we calculate the number of generated hydrocarbon molecules by multiplying the emission rate with the test period (5 min). Figure 1B shows the number of hydrocarbon molecules emitted after friction test as a function of molecular weight. The number of methane (CH_3_^+^, *m/z* = 15), ethylene (C_2_H_3_^+^; *m/z* = 27), ethane (C_2_H_5_^+^; *m/z* = 29), propane (C_3_H_7_^+^; *m/z* = 43), and butane (C_4_H_9_^+^; *m/z* = 57) molecules are 30.5 × 10^12^, 12.9 × 10^12^, 11.9 × 10^12^, 10.9 × 10^12^, and 3.6 × 10^12^, respectively. The number of generated hydrocarbon molecules decreases as the number of carbon atoms in the molecule increases, indicating that larger hydrocarbon molecules are more difficult to generate than smaller molecules. Furthermore, it is interesting to see that the number of unsaturated ethylene molecules is slightly higher than that of saturated ethane molecules, possibly because it is difficult for DLC coatings to provide sufficient hydrogen atoms for making the ethylene molecules saturated.

Here, since the carbon atoms are gradually removed atom-by-atom by the continuous hydrocarbon emission from the surfaces, we regard this continuous triboemission of hydrocarbon molecules as a chemical wear at the atomic scale for DLC. This proposed chemical wear is important because the measured rate of this chemical wear (which is experimentally measured for the first time) is about 2.332 × 10^−8^ mm^3^/Nm, which is notable and large enough compared to the conventional total wear rates of DLC. A detailed measure procedure and discussion in this part are given in text S1. Since there are few previous works reporting and quantitatively detecting the chemical wear, little is known about its underlying mechanism and how the chemical wear varies relying on the DLC structures and sliding conditions. Thus, to provide guidelines for reducing this chemical wear, we strongly require comprehensive understanding of the wear mechanisms.

### Chemical wear mechanisms

Here, we use the reactive MD simulation method to theoretically and comprehensively clarify the processes and mechanisms of the above chemical wear during the asperity collision. The simulation model is shown in fig. S2. Two DLC half cylinders are packed together with a collision depth of 1 nm, giving a realistic collision of surface asperities. The other simulation details are shown in Methods. To fully understand the chemical wear behaviors of a variety of DLCs, we prepare six DLC samples with different hydrogen concentrations because the hydrogen concentration is one of the most important factors affecting the mechanical and tribological properties of DLC (*18*, *19*). Table S1 shows the structural information for each DLC sample. The hydrogen concentrations of DLC-A, DLC-B, DLC-C, DLC-D, DLC-E, and DLC-F bulk are 0, 10, 20, 30, 40, and 50%, respectively. The DLC bulks are cleaved into half cylinders (fig. S2), and then the surfaces are fully terminated with hydrogen atoms. Friction simulations of all DLC samples are performed for 500 ps.

**Fig. 2:**
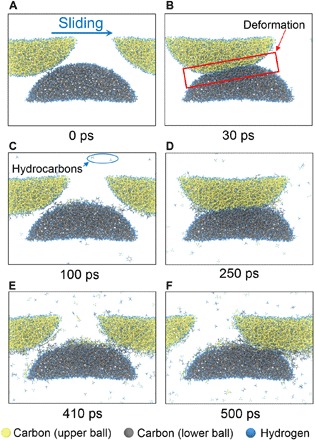
Snapshots of the friction simulation of DLC-D. (**A**) Initial configuration of sliding asperities. (**B**) Deformation of DLC asperities during the collision. (**C**) Deformation disappears during the separation of asperities, and hydrocarbon molecules are emitted from the DLC surfaces. (**D** to **F**) Continuous triboemission of hydrocarbon molecules with repeated collision and separation of sliding asperities. During the 500-ps simulation, the two DLC asperities collide five times because of the periodic boundary condition.

As a typical example of the friction simulation, Fig. 2 (A to F) shows the sliding process for DLC-D, which has a hydrogen concentration similar to that used in aforementioned experiments (Fig. 1). Figure 2A shows the initial configuration of the two DLC asperities before sliding. Two asperities collide and separate cyclically during the sliding process. The upper asperity slides in the *x* direction for one cycle of the supercell with an interval of 105.357 ps due to the periodic boundary condition. In the first friction cycle, we observe the deformation of DLC asperities near the contact region due to the collision (Fig. 2B), and subsequently, the deformation disappears gradually because of the separation of the two asperities (Fig. 2C). In addition to the substrate deformation during the sliding process, we observe the triboemission of hydrocarbon molecules (Fig. 2C), such as methane, ethane, and ethylene, generated from the friction interface, agreeing well with our friction experiments. Furthermore, as the friction simulation proceeds, more and more hydrocarbon molecules are emitted from the friction interface (Fig. 2, C to F), indicating the continuous chemical wear of DLC.

**Fig. 3:**
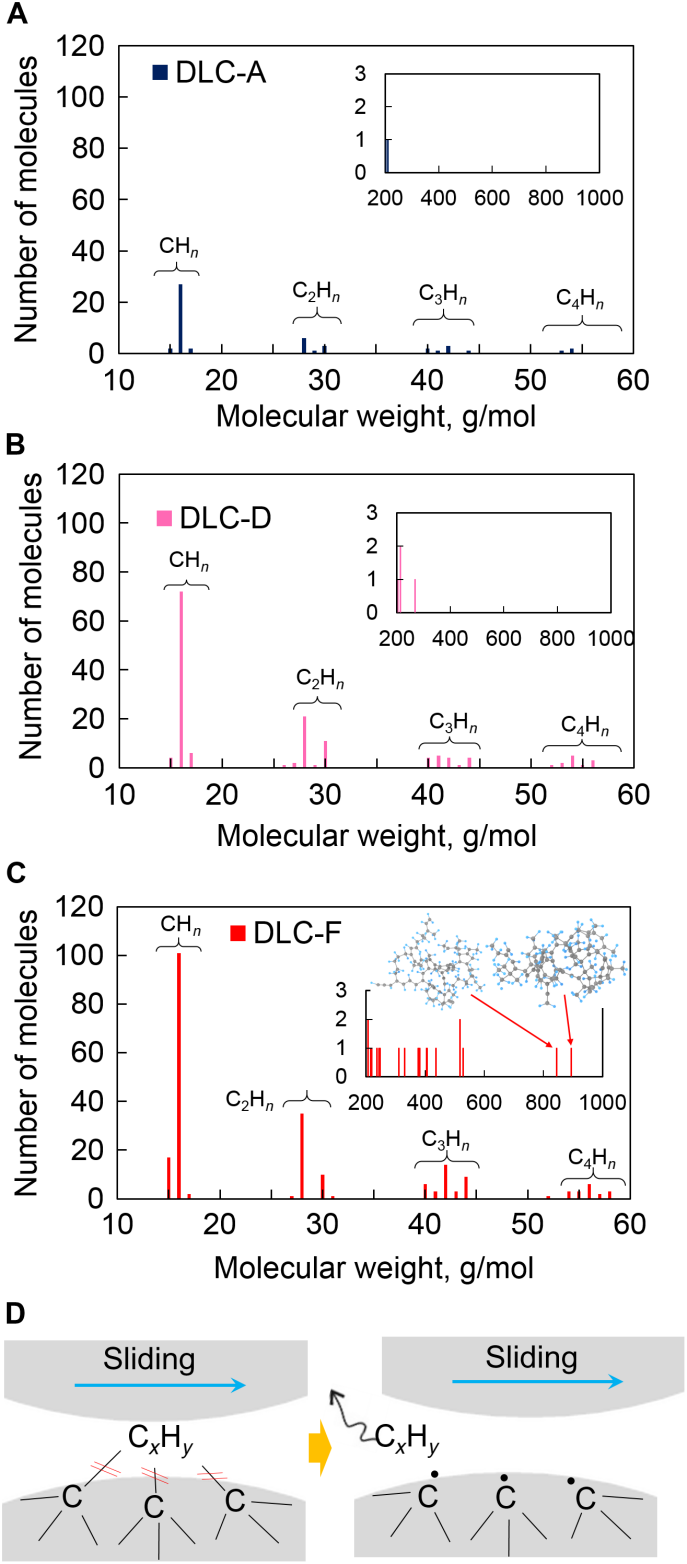
Hydrocarbon emission from DLC friction interface. Molecular weight distribution of the hydrocarbon molecules generated after friction for (**A**) DLC-A, (**B**) DLC-D, and (**C**) DLC-E, in the range of 10 to 60 g/mol. Insets in (A) to (C) show the molecular weight distributions in the range of 200 to 1000 g/mol. The inset in (C) shows snapshots of two large hydrocarbon molecules. (**D**) Schematics of the generation of a hydrocarbon molecule (C*_x_*H*_y_*). Hydrocarbon C*_x_*H*_y_* is originally connected to the DLC substrate by several C─C bonds and subsequently desorbed by the dissociation of the connective C─C bonds.

Then, to examine the chemical wear quantitatively, we survey the number and species of the emitted hydrocarbon molecules. Figure 3 (A to C) shows the molecular weight distributions of the emitted hydrocarbon molecules after friction for three typical simulations of DLC-A, DLC-D, and DLC-F, which contain 0, 30, and 50% hydrogen atoms in the bulk, respectively. The surface of DLC-A is fully terminated with hydrogen atoms, although there are no hydrogen atoms in the bulk. The chemical wear of different hydrogenated DLC can be understood through the comparison of these three cases. The molecular weight distribution results for the other DLC samples (DLC-B, DLC-C, and DLC-E) are shown in fig. S3. In Fig. 3 (A to C), the *x* axis ranges from 0 to 60 g/mol because hydrocarbons with a molecular weight of >60 g/mol are minor products (about 10.63, 12.86, and 30.39% for DLC-A, DLC-D, and DLC-F, respectively). For all the DLC samples, the major hydrocarbon molecules are small gaseous molecules, such as methane, ethane, ethylene, propane, and propylene. The number of emitted hydrocarbon molecules decreases as the number of carbon atoms in the molecule increases. This shows that the small hydrocarbon molecules are easier to generate than the large ones. On the other hand, by comparing DLC-A, DLC-D, and DLC-F, we know that the number of emitted hydrocarbon molecules for DLC-F is obviously higher than that for DLC-D, and the latter is also higher than that of DLC-A, indicating that chemical wear increases with the hydrogen concentration. We also investigate the molecular weight distribution for large hydrocarbon molecules with a molecular weight of >200 g/mol (insets in Fig. 3, A to C). In the insert graphs, only one large hydrocarbon molecule is observed for DLC-A (Fig. 3A), and three are observed for DLC-D (Fig. 3B), whereas large numbers of the large hydrocarbon molecules are emitted for DLC-F (Fig. 3C). We even observe two large hydrocarbon molecules with a molecular weight of >800 g/mol, which are considered as wear debris (inset of Fig. 3C). These above results demonstrate again that the chemical wear of DLC is accelerated with the hydrogen concentration increasing in the DLC bulk.

Next, we investigate the details of the chemical wear process and explain the severe chemical wear of highly hydrogenated DLC. Figure 3D shows schematics of the generation of hydrocarbon molecules based on the MD simulations. Initially, a hydrocarbon group (C*_x_*H*_y_*) on the surface is connected to the substrate by several bonds. During the sliding, the connective bonds between the C*_x_*H*_y_* group and DLC substrate are dissociated because of the forceful collision and deformation of the sliding asperities, and then C*_x_*H*_y_* is desorbed and emitted from the surface, resulting in the atomic-scale chemical wear. Here, we should notice that desorption of C*_x_*H*_y_* would be easier with a decreasing number of connective bonds between C*_x_*H*_y_* and the DLC substrate because fewer connective bonds are needed to dissociate. Thus, to understand why a high hydrogen concentration in DLC leads to severe chemical wear, we investigate the average number of C─C bonds that a carbon atom has in each DLC sample. This value is directly related to the number of connective bonds between C*_x_*H*_y_* and the substrate. The average numbers of C─C bonds per carbon atom for DLC-A, DLC-B, DLC-C, DLC-D, DLC-E, and DLC-F are 3.5990 ± 0.0010, 3.3919 ± 0.0602, 3.1660 ± 0.0641, 3.0713 ± 0.0422, 2.7878 ± 0.0544, and 2.5712 ± 0.0066, respectively (table S1). As the hydrogen concentration increases from 0 to 50%, the average number of C─C bonds per carbon atom decreases by roughly 30%. This indicates that there are fewer connective bonds between C*_x_*H*_y_* and the substrate with increasing hydrogen concentration; therefore, desorption and triboemission of C*_x_*H*_y_* from the highly hydrogenated DLC surface are easier than the low hydrogenated ones, leading to severe chemical wear of the highly hydrogenated DLC.

### Effect of hydrocarbon emission on the surface adhesion of DLC

In our MD simulations, in addition to the triboemission of hydrocarbon molecules, we also observe the formation of interfacial C─C bonds between two sliding asperities (Fig. 2, D to F). These interfacial C─C bonds indicate the strong adhesion of two DLC asperities, which directly determines the frictional behaviors and adhesive wear of DLC (a major type of mechanical wear) (*7*, *20*, *21*). To improve hydrogenated DLC, we need to not only reveal the intrinsic mechanisms of the chemical wear but also comprehensively understand the effects of chemical wear on other aspects of the tribological properties, especially surface adhesion. Therefore, in this section, the surface adhesion is investigated quantitatively, and we expect to find the correlation between the hydrocarbon emission and the surface adhesion.

**Fig. 4:**
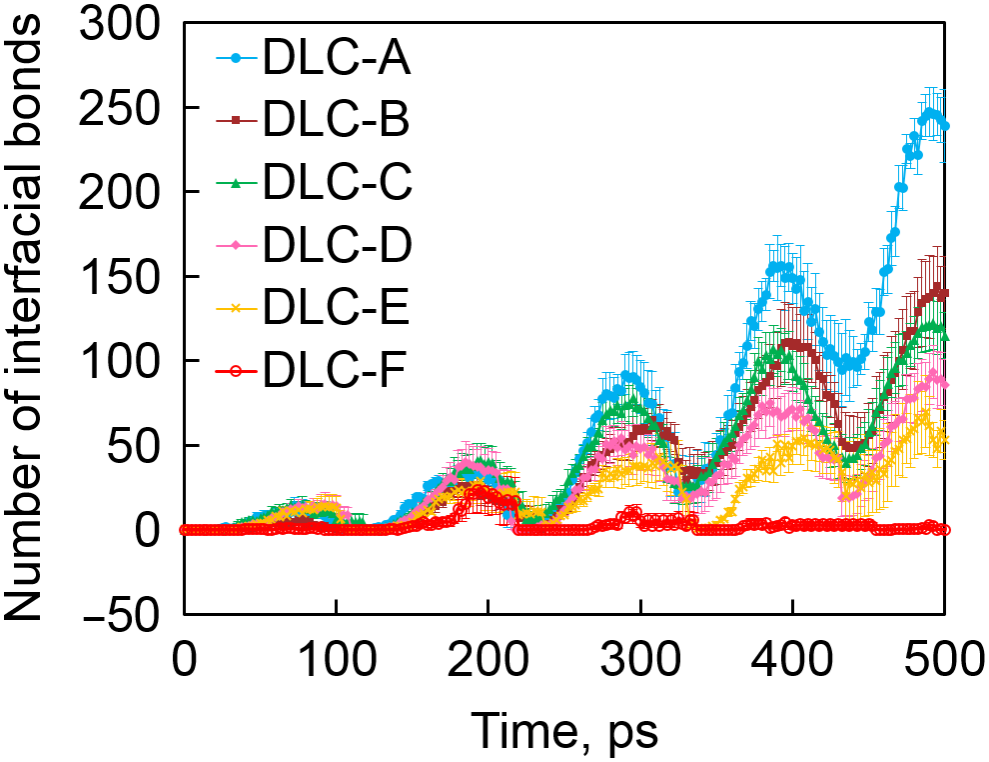
Time evolution of the number of interfacial CC bonds. ─ Blue solid circles, brown squares, green triangles, pink diamonds, orange crosses, and red open circles indicate the results for DLC-A, DLC-B, DLC-C, DLC-D, DLC-E, and DLC-F, respectively. The error bar for each sample indicates that the SE is calculated from four individual simulations with different initial structures.

Figure 4 shows the time evolution of the total number of interfacial C─C bonds at each DLC interface. For DLC-A, which contains no hydrogen atoms in the bulk (whereas the DLC-A surface is fully terminated by hydrogen), the total number of interfacial C─C bonds increases and decreases cyclically during friction because of the periodic collision and separation of the two asperities, respectively. The collision of the two asperities produces high contact pressure on the contact surface, and our previous work revealed that the high contact pressure allows interfacial C─C bonds to form (*22*). During the separation, the interfacial C─C bonds between the two asperities are stretched, and last, the bonds dissociate. Thus, the number of interfacial C─C bonds shows the periodic oscillation with repeated collision and separation of DLC asperities during friction. When the friction process proceeds, it is interesting to find that the maximum number of interfacial C─C bonds in each friction cycle increases over time, indicating an acceleration of surface adhesion. This accelerated surface adhesion is caused by the gradual removal of the terminating hydrogen atoms from the surface by the triboemission of hydrocarbon molecules during friction (Fig. 3D). The continuous hydrogen depletion produces unsaturated carbon atoms on the surface, and thus, interfacial C─C bonds are easily formed by reactions between these unsaturated carbon atoms on the surface (*9*, *21*). Thus, the triboemission of hydrocarbon molecules induces the surface adhesion during the friction. Similar periodic oscillations in the number of interfacial C─C bonds with friction cycles are observed in hydrogenated DLC-B, DLC-C, DLC-D, DLC-E, and DLC-F, although with each friction cycle there is a smaller increase in the maximum number of interfacial C─C bonds than in DLC-A. The total number of interfacial C─C bonds decreases with increasing hydrogen concentration. In particular, for DLC-F (50% hydrogen atoms in the bulk), few interfacial C─C bonds are formed during the whole friction process. These results indicate that the formation of interfacial C─C bonds is inhibited by increasing the hydrogen concentration in the DLC bulk.

This suppression of interfacial bond formation by the hydrogen atoms in the DLC bulk is due to the following mechanisms. For the highly hydrogenated DLC, although the hydrogen atoms on the surface are gradually depleted with the continuous hydrocarbon emission during friction, carbon atoms on the new surface are still terminated with hydrogen atoms that are initially in the bulk. Thus, the repulsion between terminating hydrogen atoms prevents the formation of interfacial C─C bonds. Furthermore, when the pristine terminating hydrogen atoms are depleted, we observe the gradual diffusion of hydrogen atoms from the bulk to the surface, and these diffused hydrogen atoms remove the dangling bonds and unsaturated carbon atoms on the surface, thereby suppressing the formation of the interfacial bonds. Detailed evidence and discussion of the hydrogen diffusion from the bulk to the surface are shown in text S2. Thus, overall, the increasing hydrogen concentration largely suppresses the surface adhesion of sliding DLC asperities.

### Comparison of chemical and mechanical wear of DLC

**Fig. 5:**
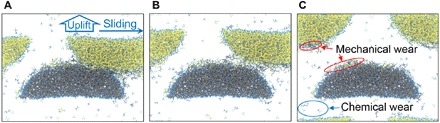
Uplift of DLC-D upper asperity. (**A**) Snapshot of DLC-D after 500-ps friction. (**B**) Upper asperity being uplifted. (**C**) Two asperities are completely separated. Chemical and mechanical wear indicated in (C) correspond to hydrocarbon emission and carbon transfer, respectively.

We further survey the adhesion-induced mechanical wear of DLC, and thus, the importance of the presently found chemical wear can be understood by comparing it with the mechanical wear. To observe the mechanical wear, the upper asperity in each simulation is lifted for 100 m/s along the *z* direction during sliding after 500 ps. Figure 5 shows the uplift of DLC-D after 500-ps friction simulation as an example. Before the uplift, the two asperities tightly adhere together (Fig. 5A). During the uplift, the interfacial bonds between the two sliding asperities are gradually stretched and dissociated (Fig. 5B). Last, two asperities are completely separated, and we observe that some of the carbon atoms in the lower (upper) DLC substrate transfer to the upper (lower) substrate (Fig. 5C). These carbon atoms transferring from their original sides to their counterparts are defined as the mechanical wear of DLC.

**Fig. 6:**
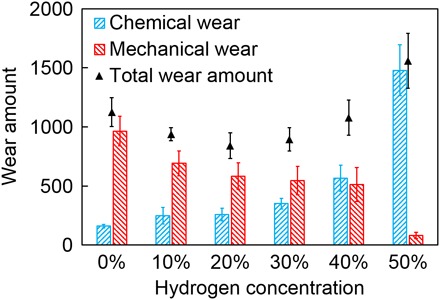
Wear amount versus hydrogen concentration. Blue and red bars indicate the amount of chemical wear and mechanical wear, respectively. Black triangles indicate the total wear amount as the sum of the chemical and mechanical wear. The error bar for each sample indicates that the SE is calculated from four individual simulations with different initial structures.

Now, we observe two kinds of atomic-scale wear of DLC: chemical and mechanical wear, which are caused by hydrocarbon emission and transfer of carbon atoms between surfaces, respectively. To compare these two types of wear quantitatively and understand their respective importance, we investigated the amounts of the two types of wear after friction. The amount of chemical wear is calculated by counting the number of carbon atoms in the emitted hydrocarbon molecules, and the amount of mechanical wear is evaluated by counting the number of carbon atoms that are transferred to their counter asperities after the uplift simulation at 500 ps. Figure 6 shows the amounts of chemical and mechanical wear for each DLC sample. For DLC-A (0% hydrogen atoms in the bulk while surfaces are terminated by hydrogen), the amount of chemical wear is small, whereas the amount of mechanical wear is very large; thus, mechanical wear is the dominant wear mechanism for DLC-A. However, as the hydrogen concentration increases, the amount of mechanical wear decreases while the amount of chemical wear increases progressively (Fig. 6), showing the converse hydrogen concentration dependences. The increasing chemical wear is caused by there being fewer connective bonds between the surface hydrocarbon group and substrate (Fig. 3D) in the highly hydrogenated DLC. For the mechanical wear, although the chemical wear–induced hydrogen depletion is accelerated by the increasing hydrogen concentration, the new surface of DLC still contains enough hydrogen terminations because they are replenished by hydrogen atoms in the bulk diffusing to the surface. Thus, the surface adhesion is suppressed by the increasing hydrogen concentration, decreasing the mechanical wear. For DLC-E (40% hydrogen atoms in the bulk), the amount of chemical wear becomes higher than the mechanical wear. By further increasing the hydrogen concentration to 50% (DLC-F), we observe a large amount of chemical wear, whereas only a small amount of mechanical wear is observed. These results indicate that the dominant wear mechanism changes from mechanical wear to chemical wear as the hydrogen concentration in the DLC bulk increases. Furthermore, we calculate the total wear amount as the sum of the chemical and mechanical wear. As the hydrogen concentration increases, the total wear amount decreases at hydrogen concentrations of 0 to 20% and increases at 20 to 50%, resulting in a concave hydrogen concentration dependence (solid triangles in Fig. 6). The total wear amount reaches its lowest value at a hydrogen concentration of around 20%, implying that this is the optimal hydrogen concentration for wear.

Besides the hydrogen concentration, we further examine how the two different types of wear vary with the sliding conditions (sliding velocity and distance). Figure S5 shows the amounts of chemical and mechanical wear of DLC-D after one friction cycle at different sliding velocities (20, 50, 100, 160, and 200 m/s). In the following, the results for mechanical and chemical wear are discussed separately to better understand their different velocity dependences. In fig. S5, the mechanical wear amount does not show obvious change as the sliding velocity increases. According to the famous Archard’s law (*23*), the adhesion-induced mechanical wear is generally proportional to the sliding distance and the applied load. Since both the sliding distance and the applied load do not change in the present simulations with different sliding velocities, the obtained mechanical wear amount does not vary with changing the sliding velocity. However, it is interesting to observe that the chemical wear amount shows a decreasing trend with the increasing sliding velocity, which is different from the mechanical wear. A full discussion on the mechanism of this interesting negative correlation between the velocity and chemical wear amount is shown in text S3. Overall, for a constant sliding distance, we demonstrate that the mechanical wear does not rely on the sliding velocity but chemical wear does. Then, in terms of the sliding distance dependence, fig. S6 shows the typical results of wear amounts as a function of sliding distance for four different DLC samples (DLC-A, DLC-C, DLC-D, and DLC-F). For DLC-A, DLC-C, and DLC-D, both chemical and mechanical wear increase almost linearly with the sliding distance, showing an Archard-like behavior. However, for DLC-F, which contains 50% hydrogen atoms in the bulk, although chemical wear still increases linearly with sliding distance, the Archard-like behavior of mechanical wear disappears because the surface adhesion is almost completely suppressed by such high hydrogen concentration (Fig. 4). The detailed discussion on this part can be found in text S4.

Moreover, we additionally use the reactive MD simulations to explore how chemical and mechanical wear are controlled by environments. In detail, we perform friction simulations by using DLC-A under a hydrogen gas environment, as shown in fig. S8A. Figure S8B shows the comparison results of chemical and mechanical wear after three friction cycles for the vacuum and the hydrogen gas environment. In the vacuum, the mechanical wear amount is much higher than the chemical wear amount, agreeing with Fig. 6; however, under a hydrogen gas environment, the mechanical wear decreases tremendously compared to the vacuum, while the chemical wear shows a neglectable change, lastly leading to a reduction of the total wear. We suppose that this hydrogen gas–induced wear reduction is because of the dissociative adsorption of hydrogen gas onto the DLC surfaces, which replenishes the lost hydrogen terminations during chemical wear. Since the surface adhesion and adhesion-induced mechanical wear is caused by the depletion of hydrogen terminations, this replenishment of hydrogen terminations can effectively suppress the surface adhesion, and last, the adhesion-induced mechanical wear is largely reduced. The above additional simulation results successfully show the possibilities of controlling chemical and mechanical wear by manipulating the environment.

## DISCUSSION

The phenomenon of triboemission has been reported by some previous studies (*24*, *25*); however, to our best knowledge, they mainly focus on the occurrence of triboemission phenomenon of the charged particles but pay little attention on the neutral molecules, and furthermore, they did not unveil how the triboemission phenomenon bridges the friction/wear behaviors. Most scientists and engineers are particularly concerned with the correlations between triboemission phenomenon and friction/wear behaviors rather than the triboemission itself. Here, we directly detect the species and amount of the triboemitted neural molecules at the DLC friction interface by both our experiments and large-scale reactive MD simulations, and furthermore, we successfully reveal how triboemission phenomenon contributes to the wear of DLC. We demonstrate that a variety of hydrocarbon molecules, mainly including methane, ethane, and ethylene, are generated and emitted from the DLC friction interface because of the forceful collision of the sliding asperities. A full discussion regarding this hydrocarbon emission mechanism can be found in text S5. The hydrocarbon emission indicates the chemical wear of DLC because the carbon atoms at the substrate are gradually removed by the continuous hydrocarbon generation reactions. Furthermore, continuous hydrocarbon emission depletes the terminating hydrogen atoms, which induces surface adhesion and adhesion-induced mechanical wear of DLC. Then, we quantitatively compare the chemical and mechanical wear to better understand the importance of chemical wear. For the DLC with low hydrogen concentrations (<30%), the amount of chemical wear is lower than mechanical wear, indicating that the mechanical wear is the dominant one. However, as the hydrogen concentration increases, the mechanical wear decreases and the chemical wear increases progressively, and thus, the dominant wear mechanism changes from mechanical to chemical wear. This result indicates that chemical wear plays a particularly important role in highly hydrogenated DLC. Moreover, our work provides an atomic-scale perspective to understand the wear behaviors of DLC. This perspective about the hydrocarbon emission and resulting chemical and mechanical wear can not only explain the complicated experimental observations of DLC but also provide useful theoretical guidance for the research on wear reduction.

For example, in an air and water environment, the wear of DLC coatings increases with increasing hydrogen concentration (*26*, *27*). This observation is explained by the fact that the surface adhesion–induced mechanical wear is largely suppressed in these water-containing environments because the water molecules passivate the unsaturated surface atoms with hydrogen and hydroxyl terminations during friction (*28*, *29*). Thus, the total wear is mainly determined by chemical wear, thereby showing a progressive increase with the hydrogen concentration. Our results also provide theoretical guidance on wear reduction. According to Fig. 6, mechanical and chemical wear are the dominant wear mechanisms for the DLC with low and high hydrogen concentrations, respectively. Thus, the wear reduction strategies should also be different depending on the hydrogen concentration of DLC. For DLC with low hydrogen concentrations, adhesion-induced mechanical wear is dominant; thus, it is essential to lower the surface adhesion for the reduction of mechanical wear, which can be achieved by introducing reactive gas such as hydrogen molecules, as we demonstrate in fig. S8. In contrast, for DLC with high hydrogen concentrations, chemical wear is dominant, and therefore, strategies for reducing the chemical wear should be a priority. On the basis of the chemical wear mechanism (Fig. 3D), increasing the number of C─C bonds in DLC substrate could effectively suppress the desorption of C*_x_*H*_y_* from the substrate and lastly lower the chemical wear. Therefore, two reasonable ways to lower the chemical wear of DLC come up: (i) decreasing the hydrogen concentration and (ii) increasing the sp^3^-hybridized carbon concentration. Decreasing the hydrogen concentration would certainly increase the number of C─C bonds in the DLC bulk and decrease the chemical wear (Fig. 6), whereas this may cause an increase in the friction coefficient (*30*, *31*). Increasing the sp^3^-hybridized carbon concentration could also increase the number of C─C bonds in the DLC bulk, and it is expected to reduce the chemical wear without compromising the low-friction properties. Thus, we consider that increasing the sp^3^-hybridized carbon concentration may be a good choice for achieving low wear of DLC. It is suggested that the DLC with high hydrogen and sp^3^-hybridized carbon concentrations would be preferred for both the low-friction and low-wear properties.

## CONCLUSION

In this study, we investigate the atomic-scale wear and the related tribochemical reactions of hydrogenated DLC by combining the advanced operando experiments and reactive MD simulations. Through the QMS analyzer placed in a vacuum chamber in which friction experiments are performed, we find a previously unidentified atomic-scale chemical wear that is induced by the continuous generation and triboemission of a variety of hydrocarbon molecules, such as methane, ethane, and ethylene, from the friction interface. Then, our reactive MD simulations reveal that the triboemission of hydrocarbon molecules is caused by the dissociation of the connective bonds between the surface hydrocarbon group and substrate during the forceful collision of surface asperities. The continuous hydrocarbon emission results in the gradual depletion of hydrogen terminations from surfaces, accelerating the surface adhesion and adhesion-induced mechanical wear of DLC. Furthermore, as the concentration of hydrogen atoms in the DLC bulk increases, the chemical wear increases progressively, whereas the mechanical wear decreases, showing the converse hydrogen concentration dependences. The increasing chemical wear originates in that carbon atoms in the DLC bulk are less bonded with each other as the hydrogen concentration increases, making the hydrocarbons easy to generate and dissociate from the friction interface. In contrast, the decreasing mechanical wear is due to the hydrogen atoms in the DLC bulk suppressing the adhesion of the sliding surfaces. The overall wear behaviors of DLC determined by both the chemical and mechanical wear show a concave hydrogen concentration dependence, giving an optimal hydrogen concentration of around 20% for achieving the best antiwear properties in DLC. In addition, the dominant wear mechanism changes from mechanical to chemical wear gradually with increasing hydrogen concentration. Thus, reducing the chemical and mechanical wear is particularly important for DLC with high and low hydrogen concentrations, respectively. This study successfully gives fundamental insights into the atomic-scale wear and related tribochemical reactions of DLC, opening a new perspective to explain the complicated experimental observations in previous studies (*26*, *27*). Moreover, as a theoretical guideline and instruction, our work contributes to the wear reduction and the improvement of the tribological properties of DLC.

## METHODS

### Experimental details

Ball-on-disk friction experiments were conducted in a high-vacuum chamber. Before the friction tests, the test chamber was heated for 6 hours at 120°C to remove water and oxygen adsorbed on the surface of the chamber. After that, the chamber was naturally cooled down to room temperature. A thermocouple was used to monitor the temperature in the chamber. The chamber was subsequently evacuated to a stable pressure of <5 × 10^−4^ Pa, and the friction test was conducted under high vacuum within the pressure range of 3.8 × 10^−4^ to 4.9 × 10^−4^ Pa. During the test, a hydrogen-free DLC-coated Si_3_N_4_ ball was rotated against a hydrogenated DLC-coated silicon wafer for 5 min at a sliding speed of 0.04 m/s. The applied load is 3 N (maximum Hertzian contact pressure of 1.05 GPa). Species of tribochemical products generated at the interface were detected with a QMS (ST-200, ULVAC Inc., Japan) in the vacuum chamber. Details of the apparatus have been described elsewhere (*15*).

The hydrogenated DLC coating on the silicon wafer is deposited using radio-frequency plasma-enhanced chemical vapor deposition with a mixture of CH_4_ and N_2_ gas. Details of the deposition parameters were reported previously (*32*). The total thickness of the hydrogenated DLC coating on the substrate is 1200 nm. The hardness and Young’s modulus measured by a nanoindentation tester are 17 and 98 GPa, respectively. The intensity ratio of the D-band (disordered) and G-band (graphitic) of the hydrogenated DLC is approximately 0.7. The composition ratios of carbon, hydrogen, and nitrogen determined by Rutherford backscattering spectroscopy are approximately 68, 31, and 1%, respectively. The hydrogen-free DLC coating on the Si_3_N_4_ ball (diameter, 8 mm) is deposited using an ion beam–assisted deposition system (IX-30-30, Hitachi, Japan) at room temperature. The total coating thickness on the substrate is 400 nm. Further information about the deposition procedures can be found in the literature (*33*, *34*). The material properties have been reported previously (*33*).

### MD simulation models

For the wear study, we use half-cylinder DLC to model the single asperity on the real surface of DLC coatings. The quick-quenching method (*7*) was used to construct the samples of DLC bulk containing different hydrogen concentrations (table S1). We cleaved the DLC bulks to form a half cylinder with a radius of 5.0 nm, and then hydrogen terminations are used to passivate the unsaturated carbon atoms on the surface. We pack two DLC asperities together with an overlap of 1.0 nm (fig. S2) to mimic real contact asperities. In the friction simulation, the top-layer atoms of the upper asperity were kept rigid and slid along the *x* direction with a velocity of 100 m/s. The bottom-layer atoms of the lower asperity were fixed during the simulation. The choice of rigid layer did not affect the tribochemical reactions and wear behaviors, as discussed in text S6. The temperature of a 0.5-nm layer neighboring the rigid and fixed layers was controlled at 300 K using the Berendsen thermostat method (*35*), and the rest of the atoms were out of the thermostat.

### MD simulation

To investigate the tribochemical reaction dynamics during friction, we used our developed MD simulator “LASKYO” (*36*) to perform the friction simulations of DLC. The bond order–dependent reactive force field (ReaxFF) (*37*) was used here to accurately handle the bond formation and dissociation during the reaction dynamics. We developed the parameters for the hydrocarbon system based on the previously reported parameter set of ReaxFF_C-2013_ (*38*). The parameter set of ReaxFF_C-2013_ provides good mechanical properties for diamond, graphite, carbon nanotubes, and amorphous carbon (*39*). Here, the parameters for the interactions of H/H and H/C are decided by the single-parameter search optimization method (*40*). The optimization details are shown in the Supplementary Materials. In our MD simulations, we used the velocity Verlet algorithm to evolve the motion of each atom with a time step of 0.25 fs per step. All the simulations were performed for 500 ps.

## Supplementary Material

http://advances.sciencemag.org/cgi/content/full/5/11/eaax9301/DC1

Download PDF

Triboemision of hydrocarbon molecules from diamond-like carbon friction interface induces atomic-scale wear

## SUPPLEMENTARY MATERIALS

Supplementary material for this article is available at http://advances.sciencemag.org/cgi/content/full/5/11/eaax9301/DC1 Text S1. Evaluation of the emission rates. Text S2. Hydrogen diffusion. Text S3. Effect of sliding velocity on the wear. Text S4. Wear amount as a function of sliding distance. Text S5. Mechanism of hydrocarbon emission. Text S6. Effect of rigid layer on the wear. Text S7. Optimization details of ReaxFF parameters. Fig. S1. Example of evaluating the evolution rate of fragment ion of CH_3_^+^. Fig. S2. Friction simulation model of DLC asperities. Fig. S3. Molecular weight distribution of the emitted hydrocarbon molecules. Fig. S4. Mean squared displacement of hydrogen atoms along *z* direction. Fig. S5. Effect of sliding velocity on the wear. Fig. S6. Wear amount as a function of sliding distance. Fig. S7. Effect of rigid layer on the wear. Fig. S8. Friction simulation of DLC-A in the hydrogen gas environment. Fig. S9. Fitting results with the first-principle calculations. Table S1. Structural information for DLC samples.
