# Supplementary material for: Triboemission of hydrocarbon molecules from diamond-like carbon friction interface induces atomic-scale wear
Source: Sci Adv. 2019 Nov 15;5(11):eaax9301. doi: 10.1126/sciadv.aax9301 (PMC6858253; doi:10.1126/sciadv.aax9301)
Supplement: http://advances.sciencemag.org/cgi/content/full/5/11/eaax9301/DC1 [file supp_5_11_eaax9301__index.html]

Science Advances | Science AdvancesAAASSearchScience AdvancesMenu

## Supplementary Materials

**This PDF file includes:**

- Text S1. Evaluation of the emission rates.
- Text S2. Hydrogen diffusion.
- Text S3. Effect of sliding velocity on the wear.
- Text S4. Wear amount as a function of sliding distance.
- Text S5. Mechanism of hydrocarbon emission.
- Text S6. Effect of rigid layer on the wear.
- Text S7. Optimization details of ReaxFF parameters.
- Fig. S1. Example of evaluating the evolution rate of fragment ion of CH3+.
- Fig. S2. Friction simulation model of DLC asperities.
- Fig. S3. Molecular weight distribution of the emitted hydrocarbon molecules.
- Fig. S4. Mean squared displacement of hydrogen atoms along *z* direction.
- Fig. S5. Effect of sliding velocity on the wear.
- Fig. S6. Wear amount as a function of sliding distance.
- Fig. S7. Effect of rigid layer on the wear.
- Fig. S8. Friction simulation of DLC-A in the hydrogen gas environment.
- Fig. S9. Fitting results with the first-principle calculations.
- Table S1. Structural information for DLC samples.

Download PDF

**Files in this Data Supplement:**

- Adobe PDF - aax9301\_SM.pdf
