# Supplementary material for: Triboemission of hydrocarbon molecules from diamond-like carbon friction interface induces atomic-scale wear
Source: Sci Adv. 2019 Nov 15;5(11):eaax9301. doi: 10.1126/sciadv.aax9301 (PMC6858253; doi:10.1126/sciadv.aax9301)
Supplement: Download PDF [file aax9301_SM.pdf]

Science Advances
AAAS

advances.sciencemag.org/cgi/content/full/5/11/eaax9301/DC1

# Supplementary Materials for

## Triboemission of hydrocarbon molecules from diamond-like carbon friction interface induces atomic-scale wear

Yang Wang, Naohiro Yamada, Jingxiang Xu, Jing Zhang, Qian Chen, Yusuke Ootani, Yuji Higuchi, Nobuki Ozawa, Maria-Isabel De Barros Bouchet, Jean Michel Martin, Shigeyuki Mori, Koshi Adachi, Momoji Kubo*

*Corresponding author. Email: momoji@imr.tohoku.ac.jp

Published 15 November 2019, *Sci. Adv.* **5**, eaax9301 (2019)
DOI: 10.1126/sciadv.aax9301

**This PDF file includes:**

Text S1. Evaluation of the emission rates.
Text S2. Hydrogen diffusion.
Text S3. Effect of sliding velocity on the wear.
Text S4. Wear amount as a function of sliding distance.
Text S5. Mechanism of hydrocarbon emission.
Text S6. Effect of rigid layer on the wear.
Text S7. Optimization details of ReaxFF parameters.
Fig. S1. Example of evaluating the evolution rate of fragment ion of $CH_3^+$.
Fig. S2. Friction simulation model of DLC asperities.
Fig. S3. Molecular weight distribution of the emitted hydrocarbon molecules.
Fig. S4. Mean squared displacement of hydrogen atoms along *z* direction.
Fig. S5. Effect of sliding velocity on the wear.
Fig. S6. Wear amount as a function of sliding distance.
Fig. S7. Effect of rigid layer on the wear.
Fig. S8. Friction simulation of DLC-A in the hydrogen gas environment.
Fig. S9. Fitting results with the first-principle calculations.
Table S1. Structural information for DLC samples.

**Text S1. Evaluation of the emission rates.**

From the intensity changes ($\Delta P$) of the fragment ions obtained by the QMS, the emission rates of the fragment ions ($R_e$) are estimated using the following equation [S. Mori, et al., *Appl. Surf. Sci.*, 27 (1987) 401-410.]

$$R_e = C\Delta P/k_B T$$

Where $C$ is the conductance ($m^3/s$) for evacuation, $k_B$ is Boltzmann's constant ($1.38\times10^{-23}$ J/K), and $T$ is the absolute temperature (K). The $\Delta P$ can be estimated from QMS analysis results as shown in Fig. 1 of manuscript. Figure S1 shows an example for calculating the $\Delta P$ for $CH_3^+$ ion. Thus, the $R_e$ of the fragment ions ($m/e$ = 15 ($CH_3^+$), 27 ($C_2H_3^+$), 29 ($C_2H_5^+$), 43 ($C_3H_7^+$) and 57 ($C_4H_9^+$)) are estimated in this study.

From the emission rate of each species of hydrocarbon molecules, we can calculate the number of emitted hydrocarbon molecules and further estimate the rate of chemical wear in our experiments. By multiplying the emission rate with the test period (5 min), the calculated number of methane ($CH_3^+$, $m/e$ = 15), ethylene ($C_2H_3^+$, $m/e$ = 27), ethane ($C_2H_5^+$, $m/e$ = 29), propane ($C_3H_7^+$, $m/e$ = 43), and butane ($C_4H_9^+$, $m/e$ = 57) molecules are $30.5 \times 10^{12}$, $12.9 \times 10^{12}$, $11.9 \times 10^{12}$, $10.9 \times 10^{12}$, and $3.6 \times 10^{12}$, respectively. Thus, if we assume that the average volume of a carbon atom is 6.7 $Å^3$ according to the density of our DLC sample, the estimated chemical wear rate is about $2.332 \times 10^{-8}$ $mm^3/Nm$.

**Text S2. Hydrogen diffusion.**

In our MD simulations, the surface hydrogen terminations are depleted away by the continuous chemical wear leaving the reactive sites like dangling bonds and unsaturated carbon atoms on the DLC surface, and thereby, the interfacial bond formation is accelerated. However, with increasing the hydrogen concentration in DLC bulk, the interfacial bond formation could be effectively suppressed. This fact indicates that we need to focus on the movements of hydrogen atoms in the DLC bulk. It is reasonable to suppose that the hydrogen atoms in the bulk diffuse to the surface when the pristine hydrogen terminations are depleted and thereby contributing to the suppression of interfacial bonds. To confirm this supposition, we investigate the mean squared displacement (MSD) of hydrogen atoms in DLC along *z*-direction. It should notice that, here we only focus on the hydrogen atoms within the collision region (about 1 nm depth as shown in fig. S2) because the MSD of hydrogen atoms in this region indicates the degree of hydrogen diffuse from the bulk to the surface.

Figure S4 shows the typical MSD results of DLC-A, -D, -E, and -F along *z*-direction. From the comparison of these four MSD results, we are able to clearly understand the difference of hydrogen movements as the hydrogen concentration changes. For DLC-A containing hydrogen atoms only on the surface, we observe the MSD along *z*-direction shows the cyclic variation until up to about 300 ps. This cyclic variation is due to the elastic deformation of DLC asperities during the repeated collision and separation as shown in Fig. 2 of manuscript. After 300 ps, the cyclic vibration of MSD disappears while shows an obvious increase. This increase in MSD along *z*-direction is most probably attributed to the mechanical wear of DLC during the sliding, that is, hydrogen atoms of the lower (or upper) substrate transfer to the upper (or lower) substrate

accompanying with the mechanical wear. For the DLC-D and DLC-E, we observe the similar vibration of MSD results as the DLC-A at about 0-300 ps. However, after about 300 ps, it is clear to see that the MSD values of DLC-E is entirely higher than DLC-D, and the latter is also slightly higher than DLC-A, indicating that the MSD of hydrogen within the collision region increases with increasing the hydrogen concentration in DLC bulk. Please notice that the mechanical wear of DLC decreases with increasing the hydrogen concentration resulting in a reducing contribution to the MSD. Therefore, the high MSD values along *z*-direction should be attributed to the diffusion of hydrogen atoms from the bulk to the collision region. Lastly, in the case of DLC-F, the MSD values increase sharply after about 200ps. Since little mechanical wear is shown (see Fig. 6 in manuscript) and the corresponding contribution to the MSD is neglectable, this sharp increase in MSD for DLC-F indicates the intense diffusion of hydrogen atoms from the bulk to the surface. Overall, based on above results, we observe the hydrogen diffusion from bulk to surface during the friction, and furthermore, the degree of diffusion increases with increasing the hydrogen concentration in the DLC bulk.

**Text S3. Effect of sliding velocity on the wear.**

Figure S5A shows the amount of chemical and mechanical wear of DLC-D after a sliding distance of 100 Å (one cycle) at different sliding velocities (20, 50, 100, 160, and 200 m/s). As the sliding velocity increases, the mechanical wear amount does not show obvious change. This result is easily to be explained by the Archard’s law which demonstrates that the adhesion-induced mechanical wear is generally proportional to the sliding distance and applied load. Since both of the sliding distance and applied load do not change in the present simulations, the mechanical wear should not vary with the sliding velocity. On the other hand, for the chemical

wear, it is interesting to observe that the wear amount shows a decreasing trend with the increasing sliding velocity (fig. S5A) which is opposite to the mechanical wear. The above results can be explained by considering that the triboemission of hydrocarbon molecules is caused by the collision and collision-induced deformation of DLC asperities as shown in the manuscript. Generally, the amount of chemical wear ($N$) is obtained by multiplying the wear rate ($r$) and contact time ($t$), that is $N = rt$. Here, as is expected normally, the chemical wear rate ($r$) defined as the number of chemically worn atoms per second increases with increasing the sliding velocity ($v$) as shown in fig. 5SB, because the high sliding velocity leads to a severe asperity collision and brings high external energy to the interface accelerating the bond dissociations in substrates. As shown in fig. S5B, the chemical wear rate is proportional to the sliding velocity, with a linear fitting of $r = 4.78 \times 10^9 v + 1.40 \times 10^{11}$. Then, in terms of the chemical wear amount ($N$) after a constant sliding distance ($d$), we have $N = (4.78 \times 10^9 + 1.40 \times 10^{11}/v)d$ derived from the relations of $N = rt$, $t = d/v$, and $r = 4.78 \times 10^9 v + 1.40 \times 10^{11}$, showing the dependences on not only the sliding distance but also the sliding velocity. Thus, for the results in fig. S5A, since the sliding distances in each simulation are unchanged, the increasing sliding velocity leads to a reduction of chemical wear amount ($N = rt$), which indicates that the decrease of contact time ($t$) is the more dominant factor than the increasing wear rate ($r$) for the chemical wear amount.

**Text S4. Wear amount as a function of sliding distance.**

Figure S6 shows the typical results of wear amounts as a function of sliding distance for four DLC samples (DLC-A, -C, -D, and -F). For DLC-A, -C, and -D, both of chemical and mechanical wear increase almost linearly with the sliding distance, showing an Archard-like

behavior. However, for DLC-F, although chemical wear still increases linearly with sliding distance, the Archard-like behavior of mechanical wear disappears because the surface adhesion is almost completely suppressed by such high hydrogen concentration. This result gives a reasonable supposition that the Archard's law may be failure to describe the conventional adhesion-induced mechanical wear for the high hydrogenated DLC. One another important message is that even for the low hydrogenated DLC (DLC-A and DLC-C) chemical wear amount at the initial two friction cycles (200 Å) is comparable or even higher than the mechanical wear, which is also an evidence that the mechanical wear is induced by the chemical wear.

**Text S5. Mechanism of hydrocarbon emission.**

Here we consider that the asperity collision and the resulted mechanical deformation of contact materials are the origin of the hydrocarbon emission, which is supported by the Fig. 1A and fig. S5B. In Fig. 1A, the hydrocarbon emission is extensive at the running-in and slows down during the steady-state period. From the running-in to steady-state period, the initial rough surfaces are planarized gradually, weakening the collision of surface asperities. Based on this correlation between the speed of hydrocarbon emission and planarization of surface asperities, we reasonably suggest that the triboemission of hydrocarbon molecules is caused by the collision of surface asperities. Furthermore, in fig. S5B, simulations show that the emission rate increases with the increasing sliding velocity. It is obvious that the high sliding velocity leads to a severe asperity collision, bringing high external energy to the interface and accelerating the bond dissociations in substrates, and consequently, the emission rate increases with the increasing sliding velocity.

**Text S6. Effect of rigid layer on the wear.**

To check the effect of rigid layers, here we perform the sliding simulations of DLC-D by changing the number of carbon atoms in the fixed layer to examine the effect of rigid layer. Figure S7 shows the amount of chemical and mechanical wear after 100 Å (one friction cycle). As we can see in fig. S7, both of the chemical and mechanical wear do not increase or decrease with increasing the number of fixed atoms in the lower substrate, although there are small variations for both wear. This result indicates that our present conclusions are not changed by the rigid layer.

**Text S7. Optimization details of ReaxFF parameters.**

The ReaxFF parameters for C/H system are determined by fitting various energies with the density functional theory (DFT) simulations. In terms of the optimization procedure, we firstly focus on the bond energies of H-H and H-C bond as shown in fig. S9A and S9B, following by the angle energies of H-H-H, H-C-H, and H-C-C as shown in figs. S9C–S9F. Then, as shown in figs. S9G and S9H, the van der Waals interaction between hydrogen atoms are also investigated because the van der Waals repulsion of hydrogen plays the key role in the frictional behavior of DLC. Lastly, in figs. S9I and S9J, we investigate the reactions of hydrogen molecule with (a) ethylene molecule and (b) diamond (100) surface, as a verification of the optimized ReaxFF parameters. For the DFT calculations of bond energy, angle energy, van der Waals, and the reaction of $H_2$/ethylene, we employ the Gaussian package with B3LYP hybrid functional and cc-pVDZ basis set; while, for the reaction of $H_2$ with diamond (100) surface, we use the $Dmol_3$ package with GGA type PBE functional and DND basis set. As the results, the optimized ReaxFF C/H parameters give the good agreement with the DFT calculations, showing the good description for the hydrocarbon system.

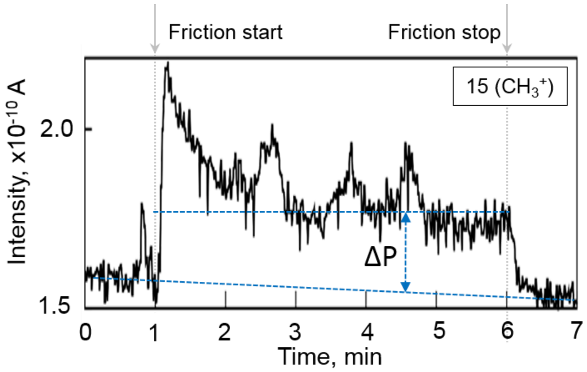


**Fig. S1. Example of evaluating the evolution rate of fragment ion of $CH_3^+$.**

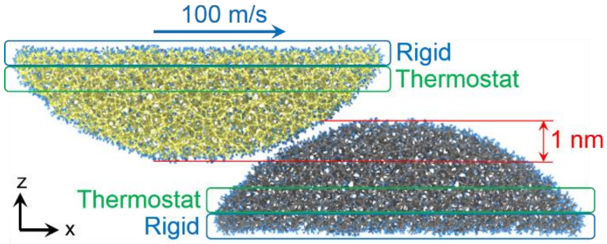


**Fig. S2. Friction simulation model of DLC asperities.** Carbon atoms in the upper asperity are shown in yellow, carbon atoms in the lower asperity are shown in gray, and all hydrogen atoms are shown in light blue

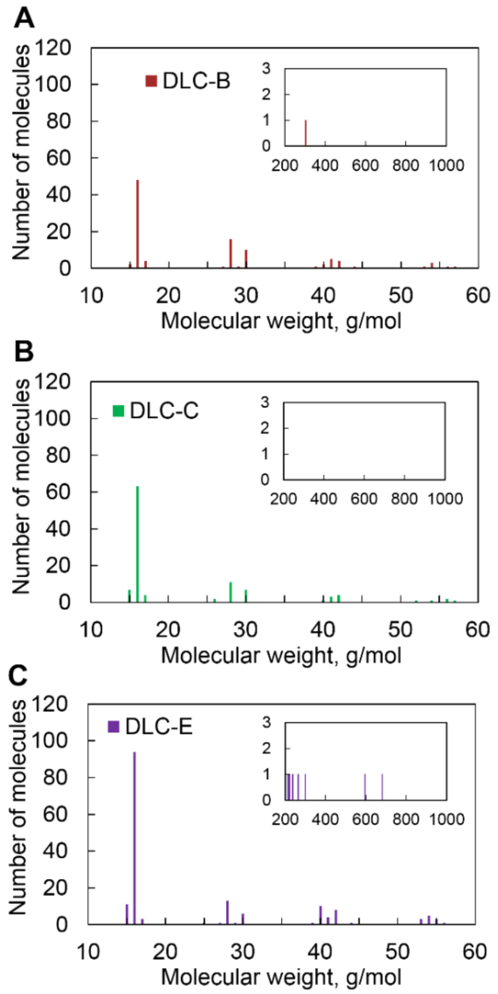


**Fig. S3. Molecular weight distribution of the emitted hydrocarbon molecules.** (**A**) DLC-B, (**B**) DLC-C, and (**C**) DLC-E contain 10%, 20%, and 40% hydrogen atoms in the bulk, respectively.

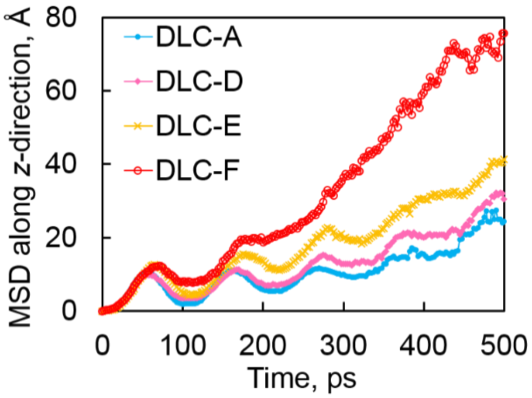


**Fig. S4. Mean squared displacement of hydrogen atoms along *z* direction.** Only the hydrogen atoms within the collision region of DLC are focsued. Blue, pink, orange, and red color indicate the MSD results for DLC-A, -D, -E, and -F, respectively.

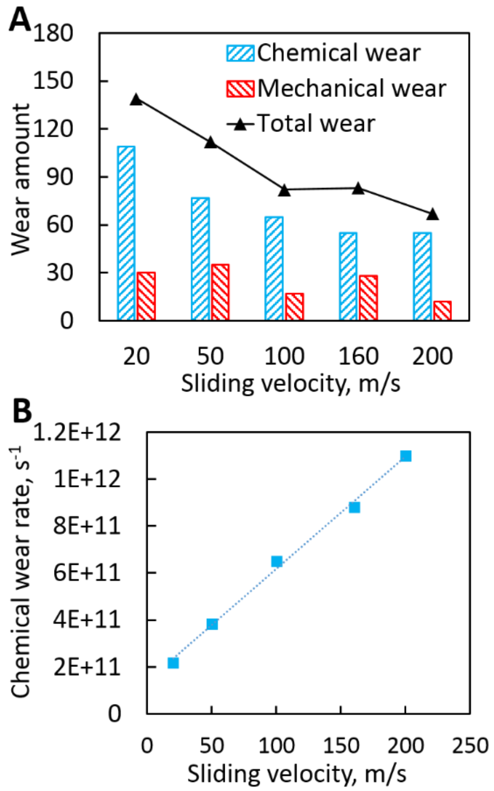


**Fig. S5. Effect of sliding velocity on the wear.** (**A**) Amount of chemical and mechanical wear of DLC-D after one friction cycle as a function of sliding velocity. (**B**) Chemical wear rate which is defined as the number of worn carbon atoms per second.

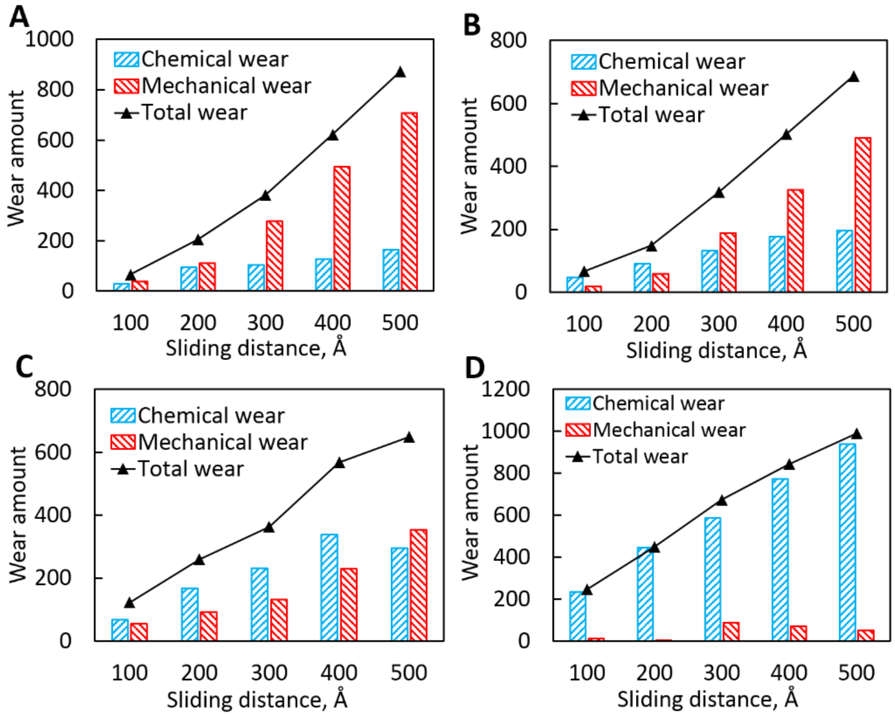


**Fig. S6. Wear amount as a function of sliding distance.** (**A**) DLC-A, (**B**) DLC-C, (**C**) DLC-D, and (**D**) DLC-F contain 0%, 20%, 30%, and 50% hydrogen atoms in the bulk, respectively.

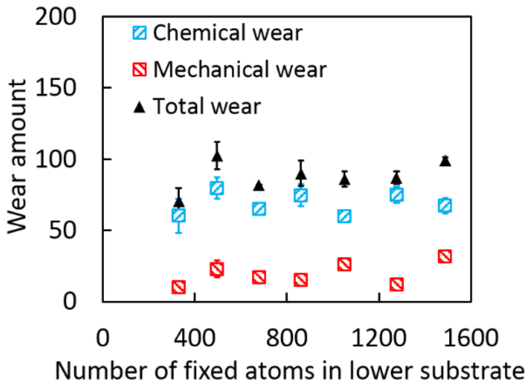


**Fig. S7. Effect of rigid layer on the wear.** Error bars are calculated from three individual simulations with different initial atomic velocities.

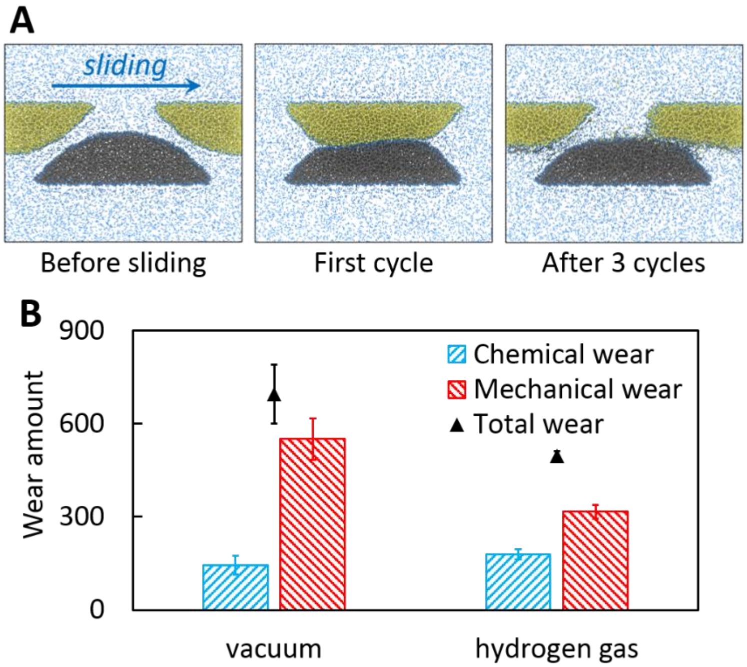


**Fig. S8. Friction simulation of DLC-A in the hydrogen gas environment.** (**A**) Typical snapshots. (**B**) Comparison of chemical and mechanical wear amount in vacuum and hydrogen gas environment. Error bars are calculated from three individual simulations with different initial atomic velocities. Pressure of hydrogen gas is 500 atm to accelerate the tribochemical reactions.

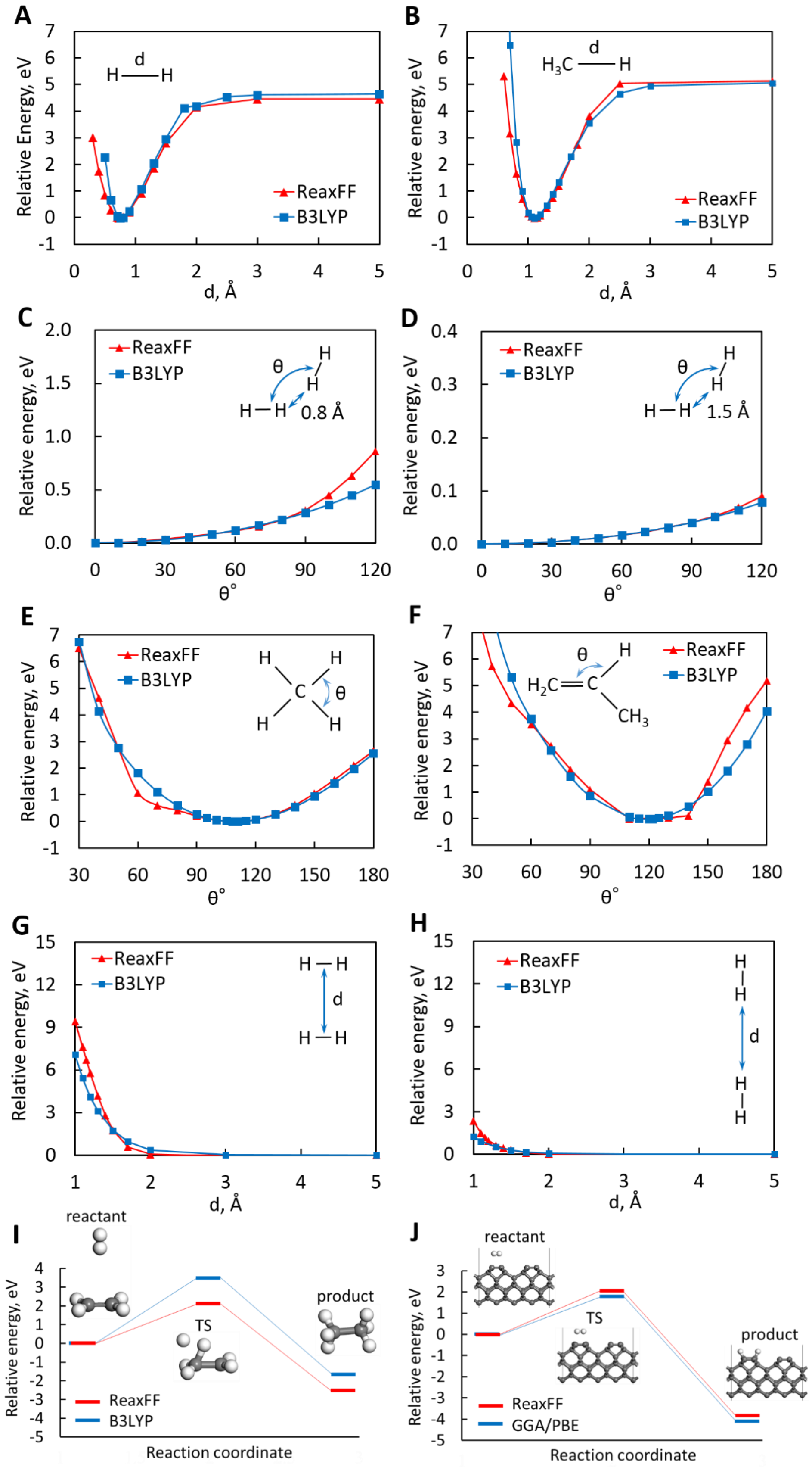
A
Relative Energy, eV
d, Å
H—H
d
ReaxFF
B3LYP
B
Relative energy, eV
d, Å
H3C—H
ReaxFF
B3LYP
C
Relative energy, eV
θ°
ReaxFF
B3LYP
θ
H—H
H
H
0.8 Å
D
Relative energy, eV
θ°
ReaxFF
B3LYP
1.5 Å
E
Relative energy, eV
θ°
ReaxFF
B3LYP
C
F
Relative energy, eV
θ°
H2C═C
CH3
ReaxFF
B3LYP
G
Relative energy, eV
d, Å
ReaxFF
B3LYP
H
Relative energy, eV
d, Å
ReaxFF
B3LYP
I
reactant
TS
product
Relative energy, eV
ReaxFF
B3LYP
Reaction coordinate
J
reactant
TS
product
Relative energy, eV
ReaxFF
GGA/PBE
Reaction coordinate

**Fig. S9. Fitting results with the first-principle calculations.** Bonding energy curves of (**A**) H-H and (**B**) H-C bond. Angle energy curves of (**C** and **D**) H-H-H, (**E**) H-C-H, and (**F**) H-C-C. van der Waals energy curves between hydrogen molecules: (**G**) two $H_2$ are placed parallel and (**H**) two $H_2$ are placed head to head. Lastly, energy barriers for desorption of hydrogen molecule: (**I**) $H_2$ reacts with ethylene molecule and (**J**) $H_2$ reacts with diamond (100) surface. TS indicates the transition state of the reaction.

**Table S1. Structural information for DLC samples.**

| Sample | H% | Density [g/cm$^3$] | Average number of C-C bonds per atom |
|---|---|---|---|
| DLC-A | 0% | 3.0825 ± 0.0037 | 3.5990 ± 0.0010 |
| DLC-B | 10% | 2.8242 ± 0.0679 | 3.3919 ± 0.0602 |
| DLC-C | 20% | 2.5548 ± 0.0660 | 3.1660 ± 0.0641 |
| DLC-D | 30% | 2.4311 ± 0.0411 | 3.0713 ± 0.0422 |
| DLC-E | 40% | 2.3117 ± 0.1448 | 2.7878 ± 0.0544 |
| DLC-F | 50% | 1.7880 ± 0.0083 | 2.5712 ± 0.0066 |
